# Elastoma: clinical and histopathological aspects of a rare disease*

**DOI:** 10.1590/abd1806-4841.20164541

**Published:** 2016

**Authors:** Marina Gagheggi Maciel, Milvia Maria Simões e Silva Enokihara, Maria Bandeira de Melo Paiva Seize, Aline Pantano Marcassi, Christiane Affonso De Donato Piazza, Silmara da Costa Pereira Cestari

**Affiliations:** 1 Universidade Federal de São Paulo (Unifesp) - São Paulo (SP), Brazil

**Keywords:** Connective tissue diseases, Elastic tissue, Nevus

## Abstract

Elastoma is a connective tissue nevus characterized by changes in elastic fibers. It can be congenital or acquired, and is usually diagnosed before puberty. Associated with osteopoikilosis, it is known as Buschke-Ollendorff syndrome. Histopathology with specific staining for elastic fibers is critical for a diagnostic conclusion. This report describes the case of a 7-year-old male patient with lesions diagnosed as elastoma, with absence of bone changes in the radiological imaging. This study aims to report the clinical presentation and histological examination of such unusual disease.

## INTRODUCTION

Elastoma was described by Weidman, Anderson, and Ayres in 1933, in a child who showed a disseminated form of the disease, hence the term juvenile elastoma.^1,2^ Also known as elastic nevus, it is part of the connective tissue nevus group (CTNs) and is characterized clinically by the formation of yellow or normochromic papules and plaques. It is typically diagnosed in children, and may be congenital or acquired, developing in the second or third decade of life. Its formation is considered an embryological dysgenesis of the dermal mesenchyme, resulting in a more prominent disorder of elastic fibers.^3^

Elastoma may be clinically associated with osteopoikilosis, which characterizes the Buschke-Ollendorff syndrome (BOS), an autosomal dominant inheritance. However, sporadic forms, unassociated with extracutaneous manifestations, have also been reported.^4^

Differential diagnosis includes other CTNs, smooth muscle hamartoma, elastofibroma, elastic pseudoxanthoma, and elastosis perforans serpiginosa.^5^

This report aims to clinically demonstrate an unusual dermatosis, and the relevance of detailed histopathology for definitive diagnosis.

## CASE REPORT

**Figure 1:**
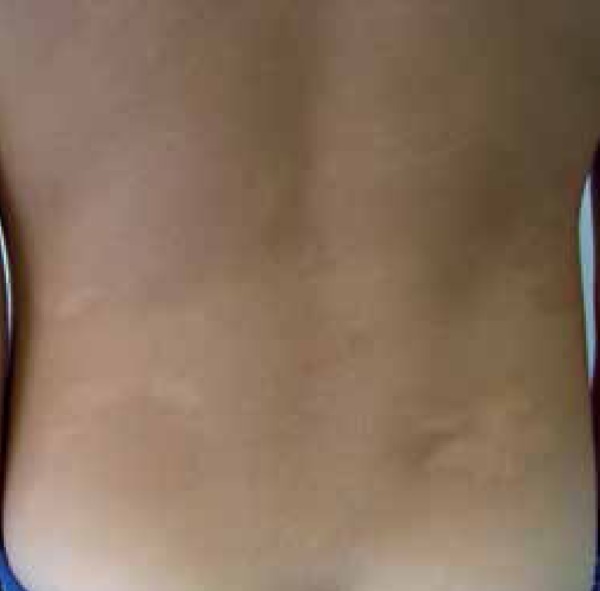
Slightly yellowish plaques on the lower back

**Figure 2:**
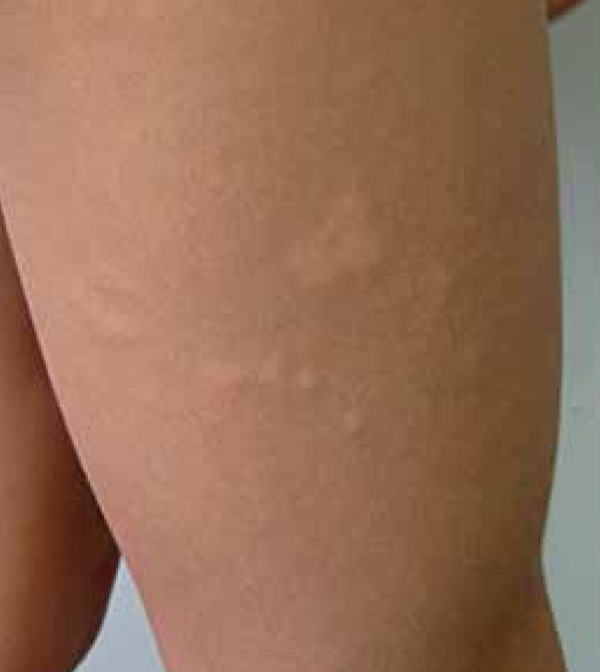
Normochromic papules and plaques on the right thigh

**Figure 3:**
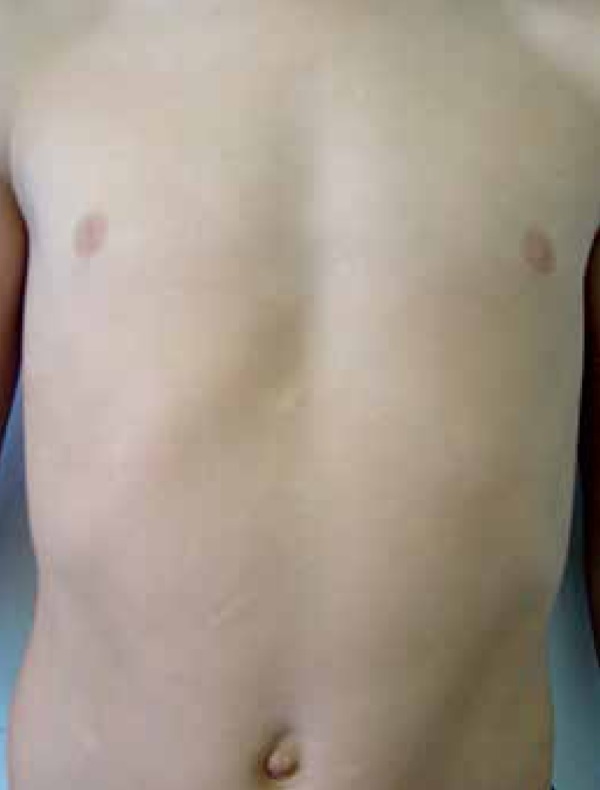
Normochromic papules and plaques on the abdomen (most recent lesions)

**Figure 4:**
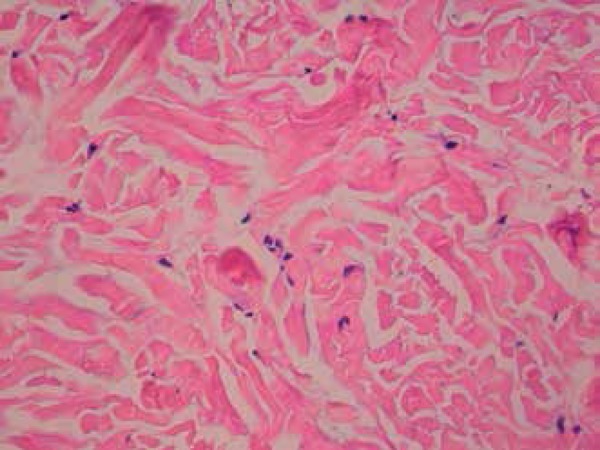
Increased spaces between the collagen fibers in the dermis (400x hematoxylin & eosin)

**Figure 5:**
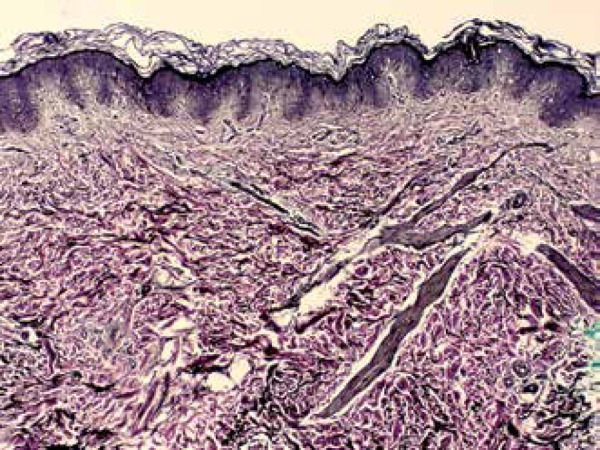
Staining of elastic fibers showing changes mainly in the middle and deep reticular dermis (100x Verhoeff)

**Figure 6:**
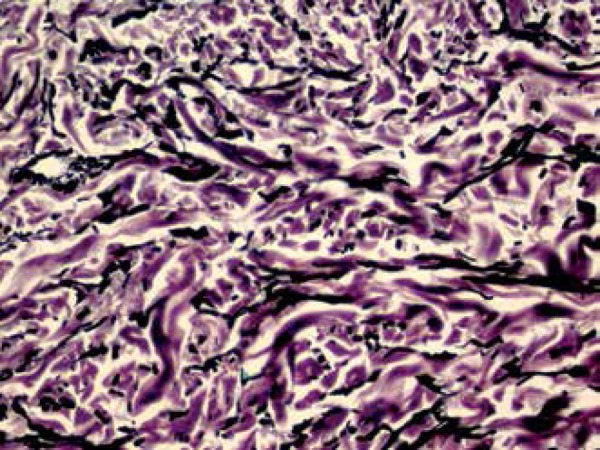
Irregular-shaped, fragmented elastic fibers (400x Verhoeff)

A 7-year-old male patient with phototype II, was brought to medical consultation by his mother, who reported the emergence of asymptomatic lesions at the age of two in the lower back, where they remained unchanged during the first three years. Two years before the consultation, the number of injuries on the lower back increased, and new lesions appeared on his lower limbs and abdomen. Dermatological examination showed normochromic oval plaques, some of which yellow, with various shapes and sizes (1 cm -10cm), spread on his lower back, anterior and lateral thigh, anterior legs, and abdomen (Figures 1-3). The patient underwent biopsy; histopathological examination with hematoxylin and eosin staining showed an increase in the spaces between collagen fibers in the dermis (Figure 4). Verhoeff's stain revealed morphological alterations in the elastic fibers, which had fragmented (elastolysis and elastorrhexis), some of them with coarser and more irregular shape than usual (Figures 5 and 6). Given the histopathological analysis, we made a clinical diagnosis of elastoma. The patient underwent radiological investigation, which was negative for bone abnormalities. The parents denied the presence of similar skin and bone changes in his family history. The patient is in outpatient follow-up and has shown the same number of lesions to date.

## DISCUSSION

CTNs are hamartomas characterized by an imbalance in the amount and distribution of collagen, elastin, and proteoglycans in the dermis.^6^

McCuaig et al. found 114 CTN cases from 1980 to 2008, 25 of which were classified as elastoma. Two of them were considered family-related, and six were caused by BOS; the remaining cases were esporadic.^7^

BOS is characterized by the presence of CTN and osteopoikilosis. Two forms of this syndrome are clinically described.^8^ The first, known as dermatofibrosis lenticularis disseminata, corresponds to a symmetrical and uniform rash of asymptomatic lichenoid papules, similar to the early phases of elastic pseudoxanthoma. The most common clinical form, in turn, shows larger, yellowish papules and nodes, sometimes grouped or coalesced, forming plaques. This rash is often asymmetric and can develop at any age, but most cases occur before puberty.

Osteopoikilosis is represented by multiple oval or rounded areas that appear opaque in bone radiography. They usually affect the bones of the carpus and tarsus, the long bones, and the pelvis. Therefore, radiological investigation should include anteroposterior X-ray of hands, wrists, feet, ankles, knees, and pelvis.

BOS is an autosomal dominant inheritance with variable expressivity, which justifies the ocurrence of both osteopoikilosis and elastoma in isolated cases. It has also been suggested that the occurrence of elastoma during adulthood might be caused by a degenerative process.^9^

In this case, the lesions are compatible with the most common clinical presentation of BOS, but the radiological study showed absence of bone changes in the child.

The alterations found in the histopathology of the elastoma are typical, regardless of whether the lesion is isolated or part of the BOS. While hematoxylin and eosin staining shows no abnormality, staining for elastic fibers show thick and tortuous bands between fairly normal collagen fibers in the reticular dermis. An increase in the number of elastic fibers around blood vessels in the papillary dermis may also be observed.^9^

It appears that the changes in the elastic fibers caused by the elastoma do not modify or increase the risk of morbidity and mortality of patients. Therefore, an accurate diagnosis of the lesions is important to reassure patients and their families, as well as to avoid unnecessary examinations and follow-up investigations. However, the skin lesions do require radiological investigation of the bones for osteopoikilosis.

Due to its rare ocurrence, there are no randomized studies assessing therapeutic possibilities for lesions diagnosed as elastoma. Therefore, there has been no known treatment to date.
